# The association between anthocyanin intake and myopia in adolescents: a cross-sectional study of NHANES

**DOI:** 10.3389/fped.2024.1503926

**Published:** 2024-11-15

**Authors:** Ying Chen, Qinglin Xu, Lu Lv, Yun Liu, Zhidan Zhang, Zhikuan Yang

**Affiliations:** ^1^ Department of Optometry and Pediatric Ophthalmology, Aier Eye Hospital, Jinan University, Guangzhou, Guangdong, China; ^2^ Department of Pediatric Ophthalmology, Aier Eye Hospital of Wuhan University (Wuhan Aier Eye Hospital), Wuhan, Hubei, China; ^3^ Department of Optometry and Pediatric Ophthalmology, Hankou Aier Eye Hospital, Wuhan, Hubei, China; ^4^ Department of Optometry and Pediatric Ophthalmology, Changsha Aier Eye Hospital, Changsha, Hunan, China

**Keywords:** adolescent, myopia, adolescents, cyanidin, petunidin, delphinidin

## Abstract

**Aim:**

The study aimed to explore the relationship of anthocyanin and its subtypes with myopia in adolescents aged 12–17 years.

**Methods:**

Adolescents data for this cross-sectional study were extracted from the National Health and Nutrition Examination Survey (NHANES) 2007–2008. Anthocyanin and subtypes were obtained using the Nutrient Database for Dietary Studies codes. Myopia was defined as a spherical equivalent of −1.0 diopters or less. The relationships between anthocyanin and subtypes intake and myopia were determined utilizing weighted univariate and multivariate logistic regression models. The relationships were also explored in gender, leisure time, physical activity, sedentary activity, BMI, and serum cotinine subgroups.

**Results:**

A total of 839 adolescents were included for further analysis, among them 245 have myopia. Malvidin (34.98%) was the subtype with the largest anthocyanin intake, followed by cyanidin (22.94%). Compared to adolescents without anthocyanin intake, total anthocyanin intake was related to a lower incidence of myopia (OR = 0.69, 95%CI: 0.51–0.92). Higher intake of cyanidin (OR = 0.69, 95%CI: 0.52–0.92), petunidin (OR = 0.64, 95%CI: 0.42–0.97), and delphinidin (OR = 0.71, 95%CI: 0.51–0.99) were associated with lower odds of myopia in adolescents. Higher total anthocyanin intake was related to lower odds of myopia in those females, leisure time physical activity ≥60 min/day, sedentary time <8 h/day, overweight or obese, and serum cotinine ≥0.05 ng/ml.

**Conclusion:**

Higher total anthocyanin intake, particularly cyanidin, petunidin, and delphinidin, was related to a lower incidence of myopia in adolescents. Increasing dietary anthocyanin intake may be an effective prevention strategy for ocular health.

## Introduction

Myopia, or nearsightedness, is a widespread visual condition marked by difficulty in seeing distant objects clearly, affecting millions of adolescents worldwide (1–3). The increasing incidence of myopia in adolescents has heightened concerns about its long-term effects on eye health and quality of life (4, 5). Myopia not only diminishes the quality of life but also creates a reliance on corrective devices like glasses or contact lenses, posing significant public health and economic challenges (6). Recent research has focused on modifiable risk factors, including dietary intake and supplementation, with particular attention to anthocyanin (7).

Anthocyanin is a subclass of flavonoids known for their vivid colors and potential health benefits. They are abundant in foods such as berries, red cabbage, and purple sweet potatoes (8). Anthocyanin plays a role in protecting against oxidative stress and inflammation, which may have potential benefits for vision and eye health (7, 9). A randomized controlled trial reported that oral consumption of 240 mg standardized bilberry extract for 12 weeks relieves the tonic accommodation of the ciliary muscle caused by visual display terminal tasks and near-vision tasks (10). The administration of anthocyanoside oligomer appears to improve visual contrast sensitivity in myopia subjects with asthenopia (11). Based on chick myopia models, compared to the control group, anthocyanin (cyanidin-3-glucoside and cyanidin-3-rutinoside) significantly reduced ocular axial length (12). Delphinidin-3-rutinoside has an inhibitory effect on bovine ciliary smooth muscle contraction (7, 13). Despite these findings, the specific relationship between anthocyanin intake and myopia in adolescents remains underexplored, warranting further investigation.

This study aims to investigate the association between anthocyanin intake, including its various subtypes, and the incidence of myopia in adolescents. Understanding this relationship could inform dietary recommendations and preventive strategies for managing myopia in adolescents, ultimately contributing to better visual health outcomes.

## Methods

### Study design and participants

Data for this cross-sectional study were extracted from the National Health and Nutrition Examination Survey (NHANES) 2007–2008. The NHANES is a program to assess the health and nutritional status of adults in the United States of America. Participants provided demographic, socioeconomic, and medical information during an in-home interview. A mobile examination center was used to conduct physical and laboratory examinations. The detailed design and data of the NHANES study are available at https://www.cdc.gov/nchs/nhanes/about_nhanes.htm.

**Figure 1:**
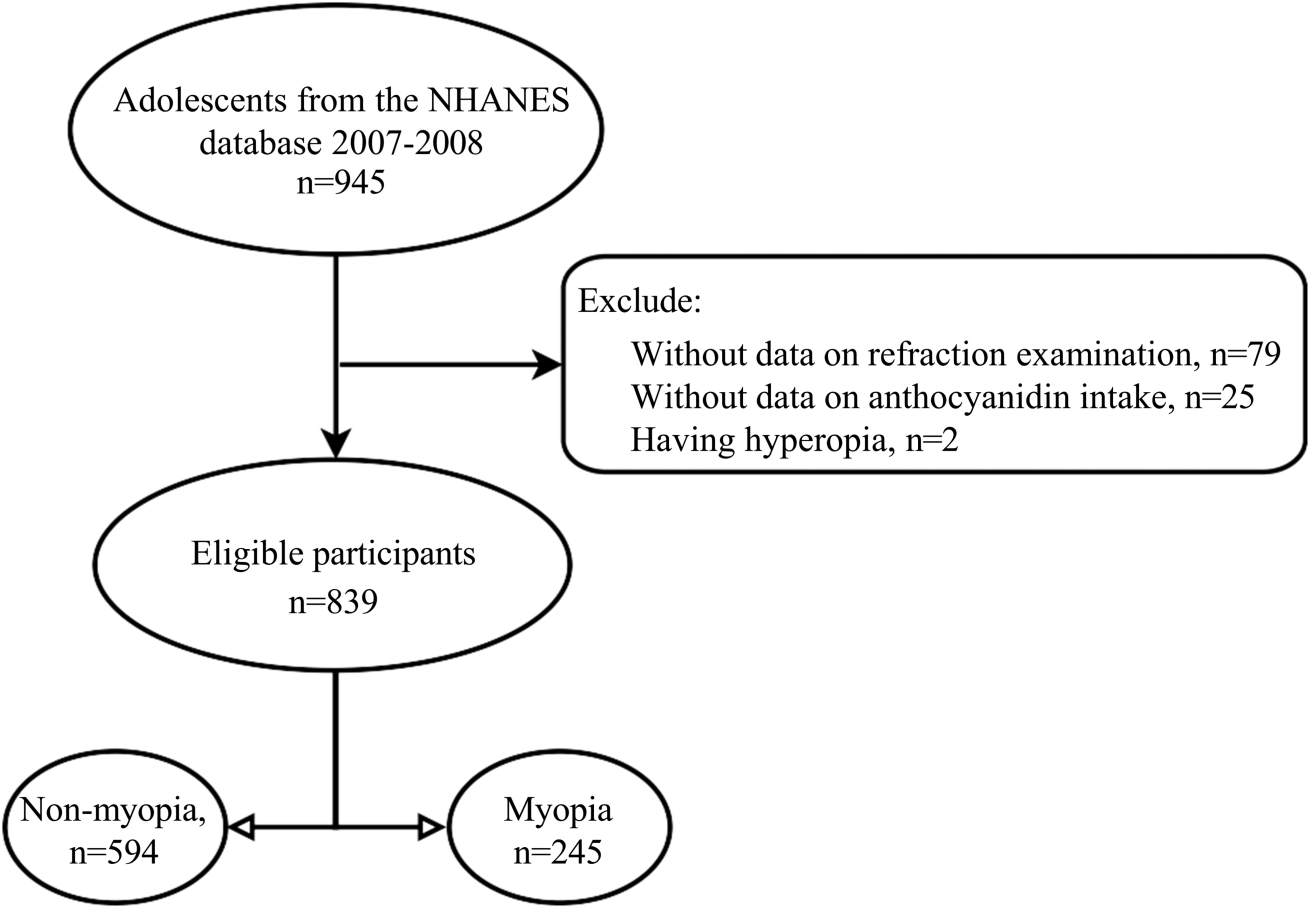
Selection process of included adolescents.

Participants would be included in those meeting the following criteria: (1) age 12–17 years old, (2) receiving a refraction examination, (3) having information on total anthocyanin intake and its sub-classes. Participants would be excluded from those having eye surgery and hyperopia. The selection process was illustrated in Figure 1. The NHANES survey protocol was approved by the National Center for Health Statistics Ethics Review Board. Thus, the Institutional Review Board's approval was exempted from the Ethics Review Board of Hospital.

### Anthocyanin intake

This study mainly evaluates anthocyanin intake from foods. Food types were refined using the Nutrient Database for Dietary Studies (FNDDS) codes, which delineate anthocyanin content. The relevant FNDDS version 4.1 was utilized for the survey cycle 2007–2008 (14). The analysis included six anthocyanin types (cyanidin, petunidin, delphinidin, malvidin, pelargonidin, and peonidin) and the total anthocyanin intake from all foods. If the total anthocyanin intake is 0 mg, it is considered as no intake, and if the intake is more than 0 mg, it is considered as intake.

### Myopia assessment

Vision examinations were performed for participants aged 12 and above. Refractive status was objectively assessed using an autorefractor (Nidek ARK-760 A, Nidek Co. Ltd., Gamagori, Japan). Each eye was measured three times consecutively, with median values for the sphere, cylinder, and axis recorded. The spherical equivalent refractive error was calculated as the sphere plus half of the column cylinder value. Myopia was defined as a spherical equivalent of −1.0 diopters or less, following previous NHANES refractive error studies (15–17).

### Covariates

Vision in adolescents may be influenced by diverse factors, so all characteristics collected in our study were adjusted (18–20). The covariates were as follows: age, gender, ethnicity (divided into Non-Hispanic White, Non-Hispanic Black, Mexican American, and Other Race), poverty income ratio (PIR), household ref person education level (including less than high school, high school or equivalent, and college or above), leisure time physical activity, sedentary activity, body mass index (BMI), serum cotinine, C-reactive protein, and total energy intake. In a typical laboratory, the level of serum cotinine and C-reactive protein were measured.

### Statistical analysis

To account for the complex sampling design of the NHANES, statistical analyses were conducted using proper weights (SDMVSTRA, SDMVPSU, WTMEC2YR) for the NHANES sampling. Continuous variables were presented as means and standard errors (S.E), while categorical variables were illustrated as numbers and percentages (%). The differences between anthocyanin intake and non-intake groups were detected utilizing *t*-tests for continuous variables, and chi-square tests for categorical variables. Weighted univariate and multivariate logistic regression models explored the association between total anthocyanin intake, its subtypes, and myopia in adolescents. The resulted were expressed as odds ratio (OR) and 95 confidence intervals (CIs). Model 1 did not adjust any covariates. Model 2 adjusted socio-demographic covariates. Model 3 adjusted all covariates. In addition, we added sensitive analysis of anthocyanin intake as continuous and categorical variables. Since adolescents with no anthocyanin intake accounted for a certain proportion, when anthocyanin was used as a categorical variable, it was classified as no intake, and the rest was divided according to the median. The results are presented in Supplementary Table S1, Figure S1. The relationships were also explored in gender, leisure time physical activity, sedentary activity, BMI, and serum cotinine subgroups. All analyses were conducted using SAS 9.4 (SAS Institute Inc., Cary, NC, USA), with two-sided *P* < 0.05 was considered statistically significant.

## Results

### Characteristics of adolescents and anthocyanin intake

**Figure 2:**
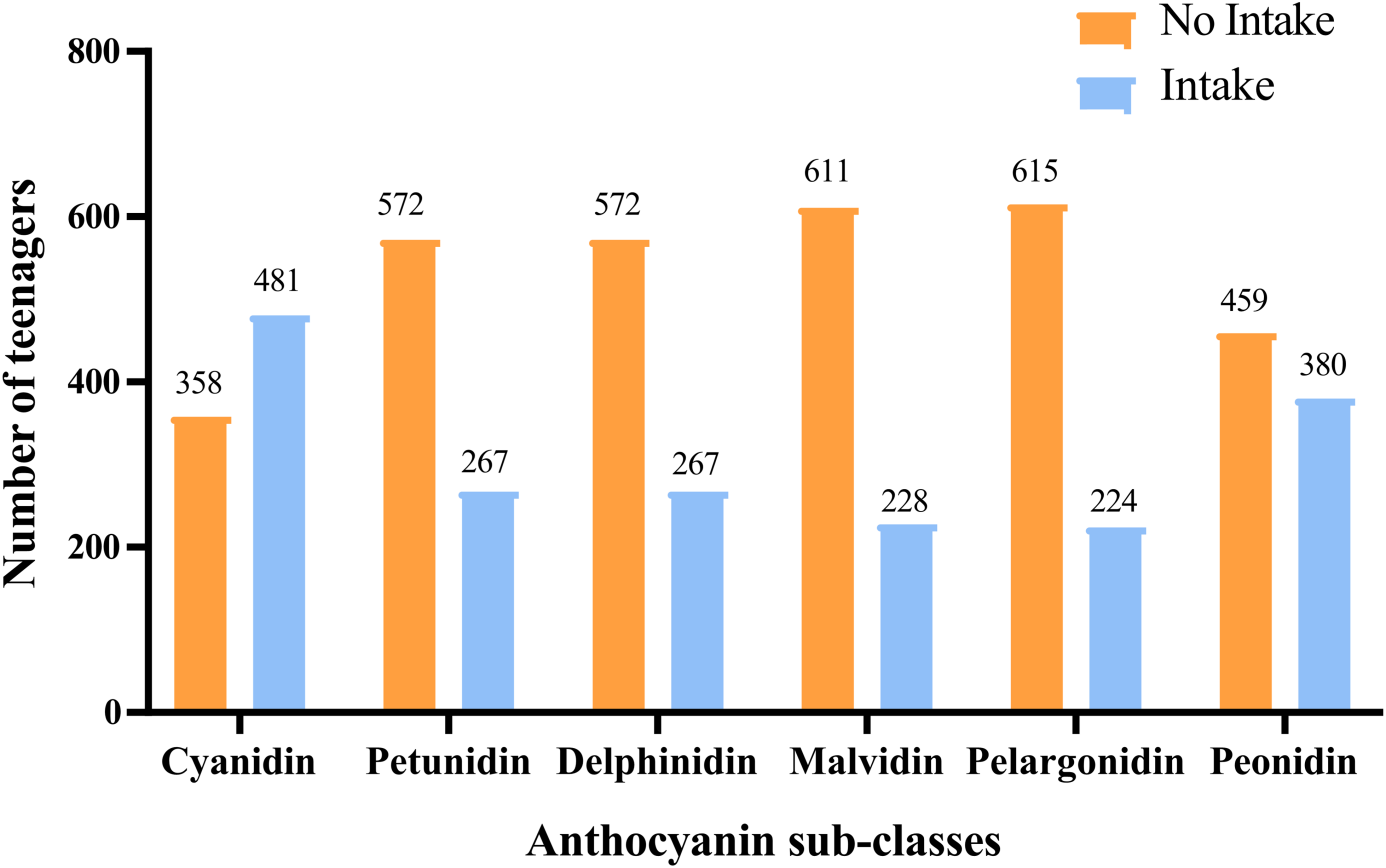
The intake of different anthocyanin subtypes intake in adolescents.

**Figure 3:**
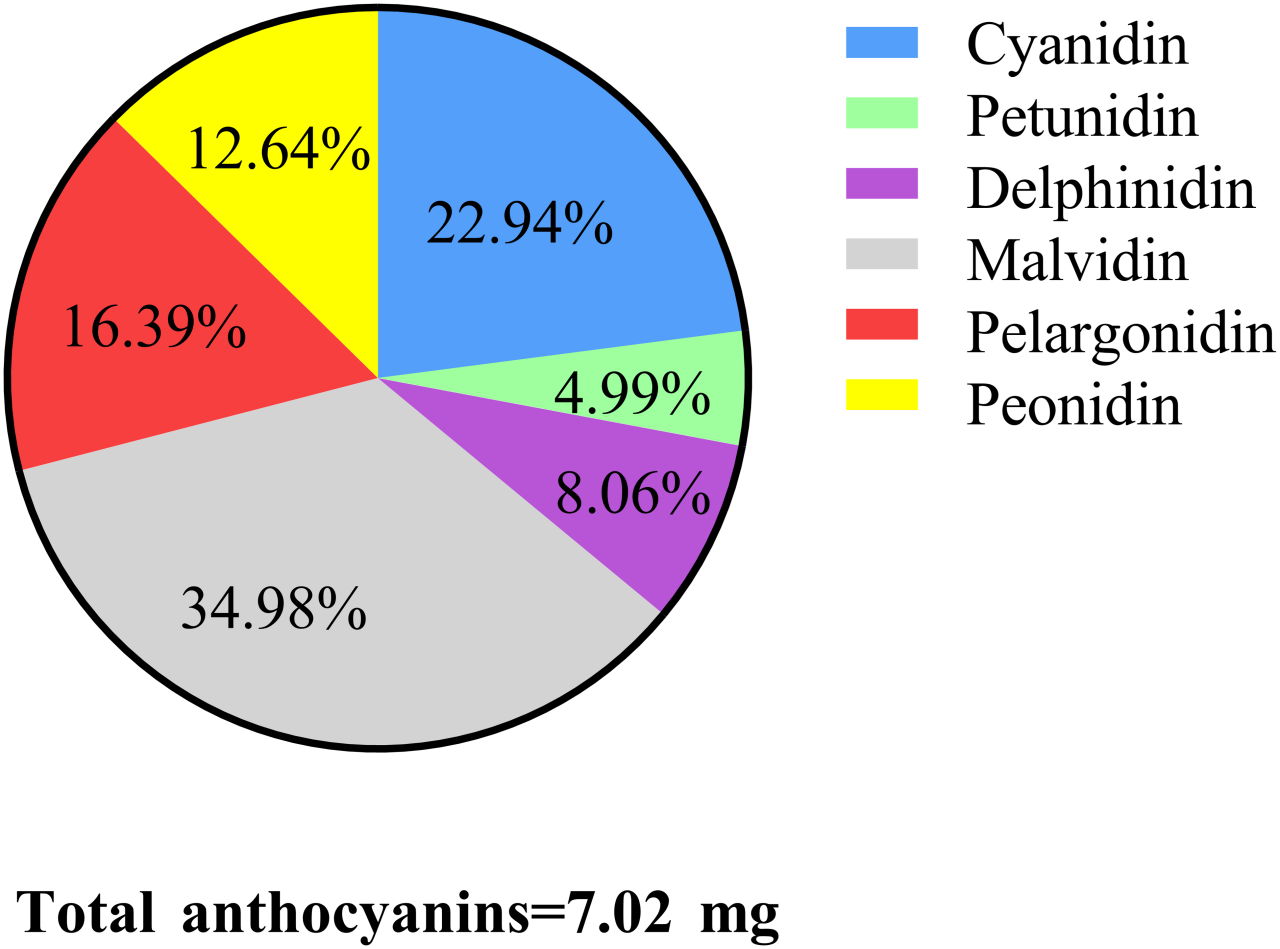
The proportion of different anthocyanin subclasses intake in adolescents.

In the study, 106 adolescents were excluded from those without information on refraction examinations (79), without data on anthocyanin intake (*n* = 25), and hyperopia (*n* = 2). A total of 839 adolescents were included for further analysis. Among them, the mean age was 14.60 (0.07) years, and 406 (50.44%) were females. 488 adolescents have anthocyanin intake. Statistical differences were observed between the two groups in ethnicity, leisure time physical activity, serum cotinine, total energy intake, and myopia (all *P* < 0.05). More detailed characteristics were presented in Table 1. Figures 2, 3 depict the intake of anthocyanin intake and its subtypes in adolescents. Malvidin (34.98%) was the subtype with the largest anthocyanin intake, followed by cyanidin (22.94%). Petunidin (4.99%) is the subtype with the lowest proportion of anthocyanin intake in adolescents.

**Table 1:** Basic characteristics of adolescents.

Variables	Total (*n* = 839)	Anthocyanin intake		*P*
		No (*n* = 351)	Yes (*n* = 488)	
Age, years, mean (S.E)	14.60 (0.07)	14.66 (0.10)	14.54 (0.10)	0.408^a^
Gender, *n* (%)				0.274^b^
Female	406 (50.44)	177 (52.90)	229 (48.42)	
Male	433 (49.56)	174 (47.10)	259 (51.58)	
Ethnicity, *n* (%)				0.035^b^
Non-Hispanic White	262 (60.36)	126 (65.77)	136 (55.93)	
Non-Hispanic Black	226 (15.23)	89 (13.03)	137 (17.04)	
Mexican American	204 (12.05)	83 (11.03)	121 (12.88)	
Other Race	147 (12.36)	53 (10.17)	94 (14.15)	
PIR, *n* (%)				0.138^b^
≤1.85	451 (40.93)	182 (37.89)	269 (43.43)	
>1.85	388 (59.07)	169 (62.11)	219 (56.57)	
Household ref person education level, *n* (%)				0.151^b^
Less than high school	264 (20.85)	107 (19.28)	157 (22.14)	
High school or equivalent	187 (21.08)	82 (24.07)	105 (18.62)	
College or above	388 (58.07)	162 (56.65)	226 (59.24)	
Leisure time physical activity, *n* (%)				0.042^b^
<60 mins/day	247 (26.33)	112 (29.73)	135 (23.54)	
≥60 mins/day	592 (73.67)	239 (70.27)	353 (76.46)	
Sedentary activity, hours, mean (S.E)	7.67 (0.30)	7.51 (0.23)	7.81 (0.43)	0.431^a^
BMI, *n* (%)				0.788^b^
Normal	485 (61.42)	216 (63.06)	269 (60.07)	
Overweight	149 (18.03)	58 (17.44)	91 (18.52)	
Obesity	205 (20.55)	77 (19.50)	128 (21.41)	
Serum cotinine, *n* (%)				0.009^b^
<0.05 ng/ml	414 (49.48)	145 (43.37)	269 (54.50)	
≥0.05 ng/ml	425 (50.52)	206 (56.63)	219 (45.50)	
C-reactive protein, mg/dl, Mean (S.E)	0.14 (0.01)	0.14 (0.01)	0.14 (0.01)	0.877^a^
Energy, kcal, mean (S.E)	2,107.84 (31.62)	1,927.43 (53.48)	2,255.89 (51.81)	0.001^a^
Myopia, *n* (%)				0.015^b^
No	594 (71.37)	239 (67.83)	355 (74.29)	
Yes	245 (28.63)	112 (32.17)	133 (25.71)	
Totals anthocyanin, mg, mean (S.E)	3.86 (0.39)	-	7.02 (0.63)	

PIR, poverty-to-income ratio; BMI, body mass index.

^a^ *t*-test.

^b^ Chi-square test; S.E, standard error.

### The association between anthocyanin intake and myopia in adolescents

The association between the intake of anthocyanin and its subtypes and myopia in adolescents was illustrated in Table 2. Compared to adolescents without anthocyanin intake, total anthocyanin intake was associated with lower incidence of myopia in model 2 (OR = 0.71, 95%CI: 0.54–0.94) and model 3 (OR = 0.69, 95%CI: 0.51–0.92). After adjusting all covariates, higher intake about subtypes of cyanidin (OR = 0.69, 95%CI: 0.52–0.92), petunidin (OR = 0.64, 95%CI: 0.42–0.97), and delphinidin (OR = 0.71, 95%CI: 0.51–0.99) were related to lower odds of myopia in adolescents. Supplementary Table S1 suggests that there is no statistically significant difference between anthocyanin intake as a continuous variable and the odds of myopia in adolescents. Grouped by median intake of anthocyanin or its subclasses, moderate intake of anthocyanin was associated with myopia in adolescents compared with no anthocyanin intake (OR = 0.60, 95%CI: 0.40–0.92), similar associations were observed in cyanidin, petunidin, and peonidin subclasses. A non-linear association between anthocyanin intake and the incidence of myopia may exist in adolescents. Supplementary Figure S1 shows the distribution of myopia prevalence in different doses of anthocyanin intake.

**Table 2:** The association between anthocyanin intake and myopia in adolescents.

Variables	Model 1		Model 2		Model 3	
	OR (95% CI)	*P*	OR (95% CI)	*P*	OR (95% CI)	*P*
Anthocyanin						
No intake	Ref		Ref		Ref	
Intake	0.73 (0.56–0.94)	0.019	0.71 (0.54–0.94)	0.020	0.69 (0.51–0.92)	0.016
Cyanidin						
No intake	Ref		Ref		Ref	
Intake	0.74 (0.56–0.96)	0.026	0.72 (0.54–0.95)	0.021	0.69 (0.52–0.92)	0.016
Petunidin						
No intake	Ref		Ref		Ref	
Intake	0.67 (0.46–0.96)	0.033	0.64 (0.43–0.95)	0.030	0.64 (0.42–0.97)	0.038
Delphinidin						
No intake	Ref		Ref		Ref	
Intake	0.71 (0.52–0.95)	0.026	0.72 (0.52–0.98)	0.040	0.71 (0.51–0.99)	0.046
Malvidin						
No intake	Ref		Ref		Ref	
Intake	0.70 (0.49–1.00)	0.053	0.71 (0.49–1.02)	0.061	0.71 (0.48–1.05)	0.078
Pelargonidin						
No intake	Ref		Ref		Ref	
Intake	0.75 (0.52–1.06)	0.099	0.71 (0.49–1.02)	0.060	0.70 (0.48–1.01)	0.056
Peonidin						
No intake	Ref		Ref		Ref	
Intake	0.82 (0.64–1.04)	0.090	0.79 (0.62–0.99)	0.042	0.77 (0.60–0.98)	0.035

OR, odds ratio; CI, confidence interval; Ref, reference. Mode1 1was univariate model. Model 2 adjusting age, gender, ethnicity, education level. Model 3 adjusting age, gender, ethnicity, household ref person education level, PIR, leisure time physical activity, sedentary activity, C-reactive protein, BMI, and serum cotinine.

### The association of anthocyanin intake with myopia in gender, leisure time physical activity, sedentary activity, BMI, and serum cotinine subgroups

The associations of anthocyanin intake with myopia were further explored in different subgroups (Table 3). Petunidin (OR = 0.60, 95%CI: 0.39–0.94) and peonidin (OR = 0.63, 95%CI: 0.43–0.94) intake were related to lower odds of myopia in males, while increased total anthocyanin (OR = 0.66, 95%CI: 0.48–0.91) and cyanidin intake (OR = 0.69, 95%CI: 0.50–0.95) were linked to lower incidence of myopia in females. In adolescents with leisure time physical activity ≥60 min/day, total anthocyanin (OR = 0.56, 95%CI: 0.39–0.81), cyanidin (OR = 0.60, 95%CI: 0.42–0.86), petunidin (OR = 0.62, 95%CI: 0.42–0.91), and peonidin (OR = 0.73, 95%CI: 0.54–0.97) intake were related to decreased odds of myopia. In adolescents with sedentary time <8 h/day, total anthocyanin (OR = 0.61, 95%CI: 0.37–0.99) and petunidin (OR = 0.55, 95%CI: 0.31–0.97) intake were related to lower occurrence of myopia. In overweight or obese adolescents, total anthocyanin (OR = 0.59, 95%CI: 0.43–0.82), cyanidin (OR = 0.61, 95%CI: 0.47–0.77), petunidin (OR = 0.60, 95%CI: 0.40–0.91), and peonidin (OR = 0.65, 95%CI: 0.45–0.95) intake were related to lower incidence of myopia. In adolescents with serum cotinine ≥0.05 ng/ml, total anthocyanin (OR = 0.46, 95%CI: 0.24–0.87) and cyanidin (OR = 0.44, 95%CI: 0.26–0.74) intake were linked to lower odds of myopia.

**Table 3:** The association between anthocyanin intake and myopia in different gender, leisure time physical activity, sedentary activity, BMI, and serum cotinine subgroups.

Subgroup	OR (95%CI)	*P*	OR (95%CI)	*P*
Gender	Male, *n* = 433		Female, *n* = 406	
No anthocyanin intake	Ref		Ref	
Anthocyanin intake	0.65 (0.39–1.09)	0.097	0.66 (0.48–0.91)	0.014
No cyanidin intake	Ref		Ref	
Cyanidin intake	0.64 (0.38–1.07)	0.086	0.69 (0.50–0.95)	0.027
No petunidin intake	Ref		Ref	
Petunidin intake	0.60 (0.39–0.94)	0.029	0.63 (0.31–1.28)	0.188
No delphinidin intake	Ref		Ref	
Delphinidin intake	0.73 (0.45–1.20)	0.199	0.59 (0.31–1.13)	0.105
No malvidin intake	Ref		Ref	
Malvidin intake	0.70 (0.45–1.11)	0.124	0.63 (0.32–1.23)	0.160
No pelargonidin intake	Ref		Ref	
Pelargonidin intake	0.59 (0.26–1.36)	0.201	0.85 (0.50–1.48)	0.550
No peonidin intake	Ref		Ref	
Peonidin intake	0.63 (0.43–0.94)	0.026	0.92 (0.65–1.28)	0.589
Leisure time physical activity	<60 mins/day, *n* = 247		≥60 mins/day, *n* = 592	
No anthocyanin intake	Ref		Ref	
Anthocyanin intake	1.23 (0.55–2.76)	0.587	0.56 (0.39–0.81)	0.004
No cyanidin intake	Ref		Ref	
Cyanidin intake	1.06 (0.48–2.32)	0.879	0.60 (0.42–0.86)	0.008
No petunidin intake	Ref		Ref	
Petunidin intake	0.76 (0.29–1.98)	0.548	0.62 (0.42–0.91)	0.018
No delphinidin intake	Ref		Ref	
Delphinidin intake	0.67 (0.27–1.71)	0.383	0.72 (0.50–1.04)	0.076
No malvidin intake	Ref		Ref	
Malvidin intake	0.72 (0.28–1.88)	0.477	0.71 (0.48–1.05)	0.079
No pelargonidin intake	Ref		Ref	
Pelargonidin intake	0.54 (0.16–1.77)	0.285	0.77 (0.51–1.16)	0.198
No peonidin intake	Ref		Ref	
Peonidin intake	1.01 (0.47–2.16)	0.976	0.73 (0.54–0.97)	0.031
Sedentary activity	<8 h, *n* = 370		≥8 h, *n* = 469	
No anthocyanin intake	Ref		Ref	
Anthocyanin intake	0.61 (0.37–0.99)	0.045	0.76 (0.52–1.12)	0.150
No cyanidin intake	Ref		Ref	
Cyanidin intake	0.64 (0.40–1.03)	0.063	0.76 (0.51–1.15)	0.181
No petunidin intake	Ref		Ref	
Petunidin intake	0.55 (0.31–0.97)	0.039	0.68 (0.41–1.14)	0.131
No delphinidin intake	Ref		Ref	
Delphinidin intake	0.77 (0.44–1.36)	0.346	0.67 (0.40–1.11)	0.112
No malvidin intake	Ref		Ref	
Malvidin intake	0.60 (0.34–1.04)	0.066	0.76 (0.46–1.24)	0.251
No pelargonidin intake	Ref		Ref	
Pelargonidin intake	0.81 (0.55–1.20)	0.267	0.63 (0.32–1.27)	0.181
No peonidin intake	Ref		Ref	
Peonidin intake	0.87 (0.61–1.23)	0.400	0.70 (0.47–1.05)	0.080
BMI	Normal, *n* = 485		Overweight or obese, *n* = 354	
No anthocyanin intake	Ref		Ref	
Anthocyanin intake	0.73 (0.48–1.11)	0.132	0.59 (0.43–0.82)	0.004
No cyanidin intake	Ref		Ref	
Cyanidin intake	0.74 (0.49–1.14)	0.159	0.61 (0.47–0.77)	<0.001
No petunidin intake	Ref		Ref	
Petunidin intake	0.62 (0.35–1.10)	0.097	0.60 (0.40–0.91)	0.018
No delphinidin intake	Ref		Ref	
Delphinidin intake	0.69 (0.47–1.03)	0.066	0.71 (0.44–1.16)	0.163
No malvidin intake	Ref		Ref	
Malvidin Intake	0.64 (0.40–1.03)	0.063	0.74 (0.45–1.21)	0.211
No pelargonidin intake	Ref		Ref	
Pelargonidin intake	0.82 (0.45–1.50)	0.498	0.55 (0.28–1.08)	0.081
No peonidin intake	Ref		Ref	
Peonidin intake	0.85 (0.57–1.26)	0.390	0.65 (0.45–0.95)	0.027
Serum cotinine	<0.05 ng/ml, *n* = 414		≥0.05 ng/ml, *n* = 425	
No anthocyanin intake	Ref		Ref	
Anthocyanin intake	0.89 (0.42–1.89)	0.754	0.46 (0.24–0.87)	0.020
No cyanidin intake	Ref		Ref	
Cyanidin intake	0.95 (0.46–1.99)	0.892	0.44 (0.26–0.74)	0.004
No petunidin intake	Ref		Ref	
Petunidin intake	0.59 (0.28–1.26)	0.160	0.64 (0.36–1.15)	0.123
No delphinidin intake	Ref		Ref	
Delphinidin intake	0.68 (0.35–1.33)	0.239	0.66 (0.37–1.18)	0.153
No malvidin intake	Ref		Ref	
Malvidin intake	0.71 (0.34–1.47)	0.337	0.65 (0.34–1.24)	0.176
No pelargonidin intake	Ref		Ref	
Pelargonidin intake	0.80 (0.40–1.59)	0.497	0.48 (0.19–1.16)	0.097
No peonidin intake	Ref		Ref	
Peonidin intake	0.83 (0.50–1.39)	0.455	0.63 (0.38–1.05)	0.072

Adjusting age, gender (not adjust gender subgroup), ethnicity, household ref person education level, PIR, leisure time physical activity (not adjust Leisure time physical activity subgroup), sedentary activity (not adjust Sedentary activity subgroup), C-reactive protein, BMI (not adjust BMI subgroup), and serum cotinine (not adjust Serum cotinine subgroup).

## Discussion

Increased anthocyanin intake is associated with a reduced incidence of myopia in adolescents aged 12–18 years. Specifically, higher overall anthocyanin intake was associated with a lower odds of developing myopia. Furthermore, when examining the effects of anthocyanin subtypes, increased intake of cyanidin, petunidin, and delphinidin was similarly linked to reduced odds of myopia. The findings suggest that dietary anthocyanins, particularly these specific subtypes, may play a role in mitigating the risk of myopia in adolescents.

The findings align with previous research that has indicated the beneficial effects of dietary antioxidants on eye health (7, 21, 22). Anthocyanins may enhance rhodopsin regeneration, thus accelerating rod cell light sensitivity (7). Fan et al. (23) also found that anthocyanin oligomer could improve dry eye disease. Cyanidin improves retinal pigment epithelium cell barrier function by reducing endoplasmic reticulum stress-induced apoptosis, with noted antioxidant activity (24, 25). Delphinidin could protect human retinal pigment epithelial cells from H_2_O_2_^−^ induced oxidative damage through anti-apoptotic and antioxidant effects (26). Our study broadens these findings to myopia, an increasingly common condition in adolescents, suggesting that anthocyanin might provide extensive ocular health benefits.

The potential mechanisms between higher anthocyanin intake and lower odds of myopia involve several biological pathways. Anthocyanin is well-recognized for its antioxidant properties, which have a vital role in mitigating oxidative stress in the retina and lens (27). The retina is particularly susceptible to oxidative damage due to its high metabolic activity and exposure to light (28, 29). Cyanidin, petunidin, and delphinidin, as specific anthocyanin subtypes, have been shown to reduce oxidative stress by scavenging free radicals and enhancing antioxidant defenses (8). The reduction in oxidative stress could potentially protect retinal cells from damage that contributes to the development of myopia. Additionally, anthocyanin has anti-inflammatory effects that may further contribute to its protective role. Chronic inflammation is known to affect ocular tissues and may influence myopia progression (30). By reducing inflammation, anthocyanin could help in mitigating the structural integrity and function of the eye. Cyanidin has been shown to inhibit pro-inflammatory cytokines and enzymes, thereby reducing inflammatory responses in various tissues (31). Finally, anthocyanins might influence ocular growth directly. They are thought to impact the regulation of eye growth through effects on signaling pathways involved in the development of myopia. Studies have shown that anthocyanin can modulate pathways such as those involving retinal pigment epithelium cells, which are crucial in maintaining proper ocular growth (32, 33). Delphinidin, in particular, has been shown to enhance blood flow to the retina, potentially supporting better ocular health and growth regulation (34).

The potential impact of anthocyanin on gut health and the microbiome should also be considered. Anthocyanins, plant-derived polyphenols, are metabolized primarily in the large intestine, where they undergo bacterial fermentation. This process not only facilitates the absorption of bioactive compounds but also influences the composition and activity of the gut microbiota (35). Increased intake of anthocyanins may lead to a greater diversity of gut bacteria, as certain strains thrive on these polyphenols and their metabolites. This modulation of gut microbiota can enhance the production of short-chain fatty acids (SCFAs), which have been shown to exhibit anti-inflammatory effects and improve gut barrier function (36). Consequently, a healthier gut microbiome may contribute to improved systemic health, potentially influencing ocular development and reducing the risk of myopia. Conversely, an alteration in gut microbiome composition due to excessive anthocyanin metabolism could lead to imbalances, promoting the growth of pathogenic bacteria and resulting in dysbiosis. Dysbiosis has been linked to various health issues, including inflammation and metabolic disorders, which may indirectly affect ocular health (37).

The differences in anthocyanin intake and myopia between males and females may reflect underlying physiological and hormonal variations. In males, the association of higher petunidin and peonidin intake with reduced myopia odds might be attributed to these compounds’ specific effects on retinal health and visual function, potentially mediated by gender-specific metabolic pathways or hormonal influences. In females, the relationship between higher total anthocyanin and cyanidin intake and lower myopia odds suggests that these compounds may more broadly influence ocular health. The association between higher anthocyanin intake and reduced incidence of myopia in adolescents with leisure time physical activity ≥60 min/day, indicates the potential synergistic effects of diet and physical activity on ocular health. The additional intake of anthocyanin could amplify these beneficial effects. Obesity is often linked to increased systemic inflammation and oxidative stress, which are known risk factors for myopia (3). Anthocyanin, such as cyanidin and petunidin, has shown potential in reducing oxidative stress and inflammation, thus potentially counteracting the negative impact of excess weight on ocular health (7, 38). Similarly, anthocyanin could attenuate smoking-induced acute endothelial dysfunction and improve peripheral temperature in young smokers (39).

Dietary interventions may as a preventive measure against myopia in adolescents. Given the rising prevalence of myopia in younger populations, incorporating anthocyanin-rich foods into the diet could offer a cost-effective and non-invasive strategy to mitigate the risk of developing myopia. Nutritional recommendations emphasizing foods rich in anthocyanin, such as berries and certain vegetables, could be beneficial as part of broader public health initiatives aimed at reducing myopia rates among adolescents. It is important to consider the anthocyanin content in these foods alongside realistic intake levels and absorption rates to ensure effective protective benefits. For instance, typical servings of these fruits and vegetables may yield varying amounts of anthocyanins, which may influence their overall efficacy. Future studies should focus on establishing specific intake targets that reflect not only the anthocyanin composition of these foods but also the bioavailability and potential protective mechanism of these compounds in relation to myopia development. By understanding these factors, we can provide more precise and actionable dietary guidelines that can be realistically adopted in everyday diets, thus maximizing their potential health benefits for adolescents.

Nevertheless, the study has several limitations. First, the research utilized a public dataset with a cross-sectional design, preventing the establishment of a causal relationship between anthocyanin intake and myopia in adolescents. Second, although many covariates were adjusted for, unmeasured covariates in the database may influence our findings. Third, since anthocyanin intake reflects only the current status, and lack of validation studies specifically related to anthocyanin intake assessment, prospective cohort studies are needed to explore its association with myopia in adolescents. Finally, the current analysis is not based on direct measurements of anthocyanin levels in blood or urine, but rather on estimates derived from dietary data. More objective measurements of anthocyanin intake should be investigated in the future.

## Conclusion

Higher anthocyanin intake, particularly cyanidin, petunidin, and delphinidin, was associated with a lower incidence of myopia in adolescents. The findings support the potential for dietary anthocyanin to contribute to myopia prevention strategies. Future research should focus on longitudinal studies and clinical trials to validate these findings and further explore the underlying mechanisms of anthocyanins’ effects on ocular health.

## Data Availability

Publicly available datasets were analyzed in this study. This data can be found here: NHANES, https://www.cdc.gov/nchs/nhanes/about_nhanes.htm.
