# Supplementary material for: The association between anthocyanin intake and myopia in adolescents: a cross-sectional study of NHANES
Source: Front Pediatr. 2024 Nov 15;12:1503926. doi: 10.3389/fped.2024.1503926 (PMC11604415; doi:10.3389/fped.2024.1503926)
Supplement: Supplementary file 1 [file Datasheet1.docx]

Table S1 The association between anthocyanin intake and myopia in adolescents

| Variables (mg) | Model 1 |  | Model 2 |  |
| --- | --- | --- | --- | --- |
|  | OR (95%CI) | *P* | OR (95%CI) | *P* |
| Anthocyanin | 1.00 (0.99-1.01) | 0.556 | 1.00 (1.00-1.01) | 0.526 |
| Cyanidin | 1.03 (0.98-1.09) | 0.225 | 1.03 (0.97-1.10) | 0.284 |
| Petunidin | 1.08 (0.90-1.29) | 0.389 | 1.07 (0.89-1.27) | 0.453 |
| Delphinidin | 1.00 (0.89-1.12) | 0.946 | 1.00 (0.89-1.11) | 0.930 |
| Malvidin (mg) | 1.00 (0.98-1.02) | 0.898 | 1.00 (0.98-1.02) | 0.910 |
| Pelargonidin | 0.98 (0.92-1.05) | 0.567 | 0.98 (0.92-1.04) | 0.503 |
| Peonidin | 1.00 (0.99-1.01) | 0.718 | 1.00 (0.99-1.02) | 0.674 |
| Anthocyanin |  |  |  |  |
| No intake | Ref |  | Ref |  |
| ≤2.36 | 0.61 (0.41-0.92) | 0.020 | 0.60 (0.40-0.92) | 0.021 |
| >2.36 | 0.85 (0.59-1.24) | 0.384 | 0.82 (0.56-1.22) | 0.306 |
| Cyanidin |  |  |  |  |
| No intake | Ref |  | Ref |  |
| ≤0.49 | 0.68 (0.46-0.99) | 0.045 | 0.67 (0.46-0.97) | 0.037 |
| >0.49 | 0.80 (0.54-1.19) | 0.246 | 0.77 (0.52-1.14) | 0.170 |
| Petunidin |  |  |  |  |
| No intake | Ref |  | Ref |  |
| ≤0.20 | 0.55 (0.35-0.87) | 0.014 | 0.51 (0.32-0.82) | 0.009 |
| >0.20 | 0.80 (0.51-1.25) | 0.303 | 0.80 (0.50-1.27) | 0.321 |
| Delphinidin |  |  |  |  |
| No intake | Ref |  | Ref |  |
| ≤0.26 | 0.60 (0.36-0.97) | 0.040 | 0.60 (0.35-1.03) | 0.062 |
| >0.26 | 0.83 (0.52-1.32) | 0.401 | 0.84 (0.53-1.33) | 0.427 |
| Malvidin |  |  |  |  |
| No intake | Ref |  | Ref |  |
| ≤0.91 | 0.60 (0.34-1.07) | 0.081 | 0.61 (0.33-1.11) | 0.101 |
| >0.91 | 0.81 (0.46-1.43) | 0.443 | 0.82 (0.47-1.42) | 0.448 |
| Pelargonidin |  |  |  |  |
| No intake | Ref |  | Ref |  |
| ≤0.19 | 0.66 (0.40-1.07) | 0.085 | 0.63 (0.39-1.02) | 0.060 |
| >0.19 | 0.84 (0.48-1.46) | 0.507 | 0.78 (0.44-1.40) | 0.388 |
| Peonidin |  |  |  |  |
| No intake | Ref |  | Ref |  |
| ≤0.20 | 0.77 (0.56-1.06) | 0.099 | 0.72 (0.55-0.96) | 0.028 |
| >0.20 | 0.87 (0.57-1.31) | 0.471 | 0.85 (0.57-1.27) | 0.404 |

OR: odds ratio; CI: confidence interval; Ref: reference

Mode1 1: Univariate model,

Model 2 adjusting age, gender, race/ethnicity, and education level.

There are not enough degrees of freedom for Model 3.


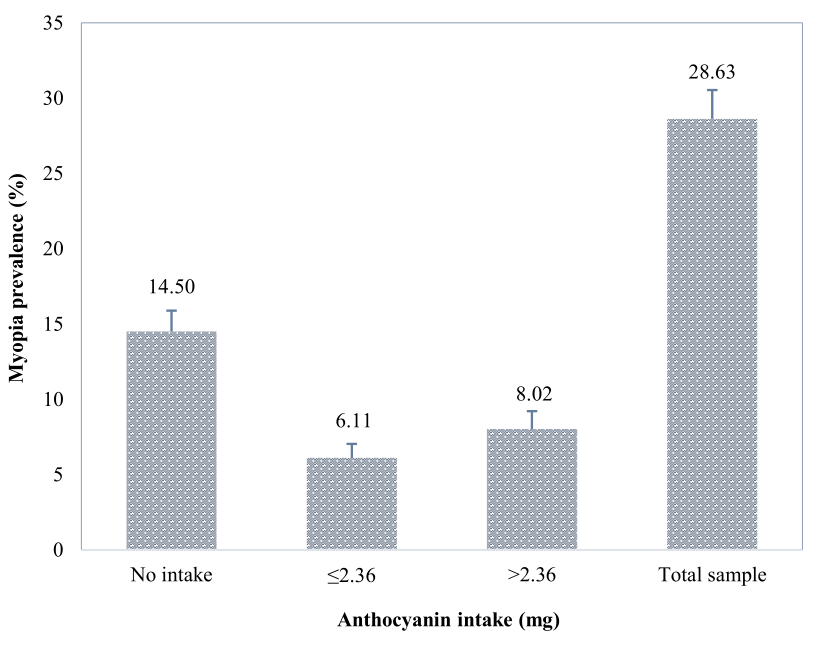


Figure S1 Distribution of myopia prevalence in different doses of anthocyanins
