# Supplementary figures and images for: The association between anthocyanin intake and myopia in adolescents: a cross-sectional study of NHANES
Source: Front Pediatr. 2024 Nov 15;12:1503926. doi: 10.3389/fped.2024.1503926 (PMC11604415; doi:10.3389/fped.2024.1503926)

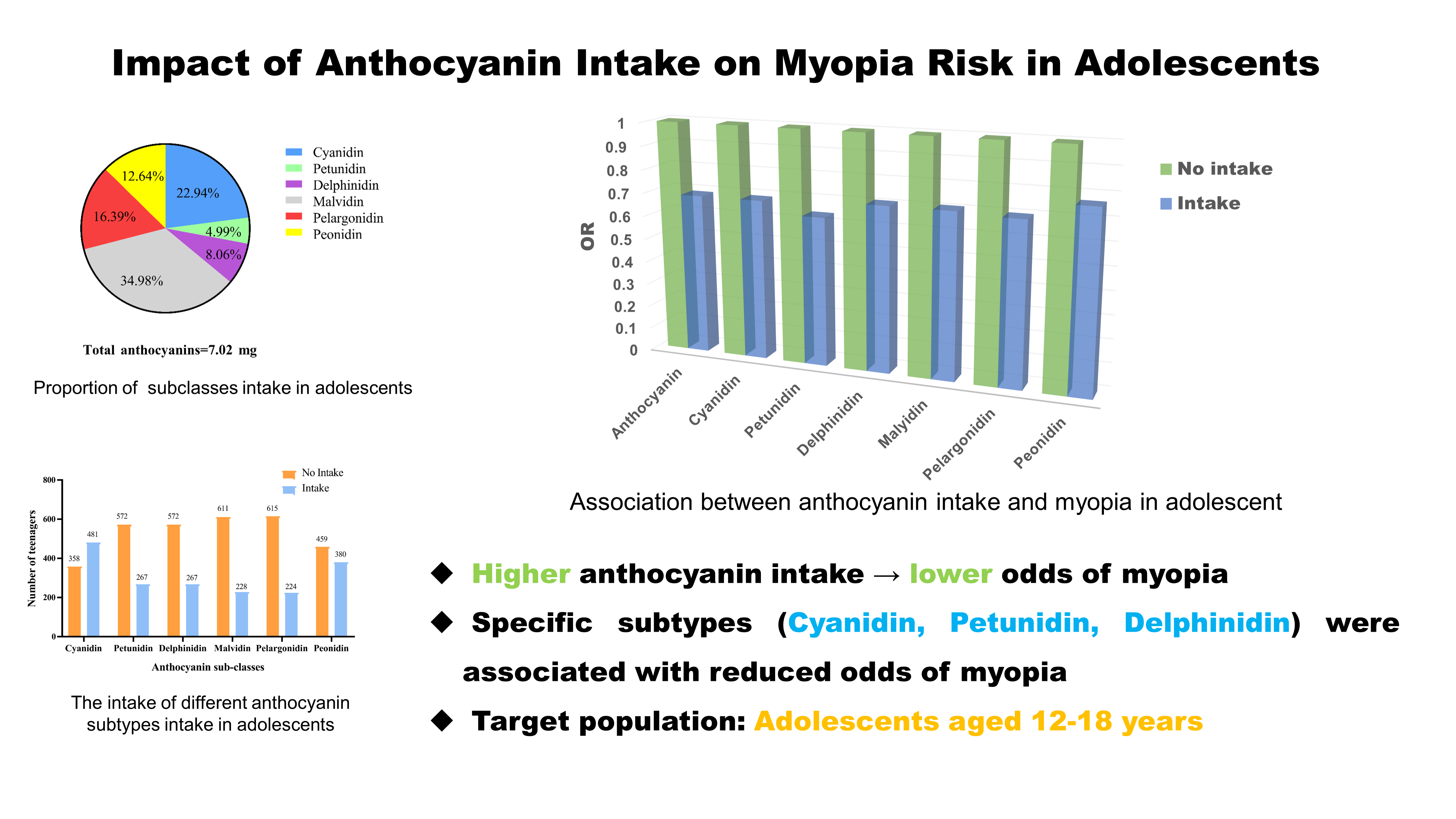

Supplement: Supplementary file 2 [file Image1.tif]
